# Prognostic nomogram for patients with hepatocellular carcinoma underwent adjuvant transarterial chemoembolization following curative resection

**DOI:** 10.1097/MD.0000000000006140

**Published:** 2017-03-24

**Authors:** Chu-Yu Jing, Yi-Peng Fu, Su-Su Zheng, Yong Yi, Hu-Jia Shen, Jin-Long Huang, Xin Xu, Jia-Jia Lin, Jian Zhou, Jia Fan, Zheng-Gang Ren, Shuang-Jian Qiu, Bo-Heng Zhang

**Affiliations:** The Liver Cancer Institute, Zhongshan Hospital and Shanghai Medical School, Key Laboratory for Carcinogenesis and Cancer Invasion, The Chinese Ministry of Education, Shanghai, P.R. China.

**Keywords:** hepatocellular carcinoma, nomogram, prognosis, transarterial chemoembolization

## Abstract

Adjuvant transarterial chemoembolization (TACE) is a major option for postoperative hepatocellular carcinoma (HCC) patients with recurrence risk factors. However, individualized predictive models for subgroup of these patients are limited. This study aimed to develop a prognostic nomogram for patients with HCC underwent adjuvant TACE following curative resection.

A cohort comprising 144 HCC patients who received adjuvant TACE following curative resection in the Zhongshan Hospital were analyzed. The nomogram was formulated based on independent prognostic indicators for overall survival (OS). The performance of the nomogram was evaluated by the concordance index (C-index), calibration curve, and decision curve analysis (DCA) and compared with the conventional staging systems. The results were validated in an independent cohort of 86 patients with the same inclusion criteria.

Serum alpha-fetoprotein (AFP), hyper-sensitive C-reactive protein (hs-CRP), incomplete tumor encapsulation, and double positive staining of Cytokeratin 7 and Cytokeratin 19 on tumor cells were identified as independent predictors for OS. The C-indices of the nomogram for OS prediction in the training cohort and validation cohort were 0.787 (95%CI 0.775–0.799) and 0.714 (95%CI 0.695–0.733), respectively. In both the training and validation cohorts, the calibration plot showed good consistency between the nomogram-predicted and the observed survival. Furthermore, the established nomogram was superior to the conventional staging systems in terms of C-index and clinical net benefit on DCA.

The proposed nomogram provided an accurate prediction on risk stratification for HCC patients underwent adjuvant TACE following curative resection.

## Abstract 2

Supplemental Digital Content is available in the text

## 1 Introduction

Hepatocellular carcinoma (HCC) is the fifth most common and the second most lethal cancer worldwide, with China alone accounts for over 50% of all cases and deaths.^[1]^ In historically low-risk areas, including the United States and Western Europe, the incidence of HCC is increasing as well.^[1]^ The prognosis of the curative treatments, among which resection is the first option,^[2]^ is jeopardized by a high recurrence rate.^[3]^ Hence, HCC remains one of the most lethal malignancies and poses a great therapeutic challenge.

Adjuvant therapies after curative resection that can substantially lower postoperative recurrence risk are urgently needed. However, adjuvant therapies including transarterial chemoembolization (TACE),^[4]^ adoptive immunotherapy,^[5,6]^ sorafinib,^[7]^ and interferon therapy^[8]^ showed inconsistent efficacies and standard postoperative adjuvant therapies for HCC remained unestablished.

TACE, recommended as first-line therapy in unresectable HCC,^[9]^ yielded conflicting results as an postoperative adjuvant therapy for HCC.^[4,10,11]^ A meta-analysis reviewed 6 randomized control trials (RCT) and demonstrated adjuvant TACE improved survival in HCC patients with tumor size > 5 cm or vascular invasion.^[4]^ However, in a retrospective propensity score analysis based on an HCC cohort without satellite nodules and vascular invasion, adjuvant TACE showed no superiority in terms of the survival rate and the recurrence rate.^[11]^ Although no large-scale, multi-centered RCTs were available to address the issue, several studies based on HCC patients with more tumor numbers, larger tumor size, and vascular invasion were in favor of adjuvant TACE.^[10,12–15]^ Therefore, adjuvant TACE (1–3 courses) is widely used in postoperative HCC patients with a range of recurrence risk factors such as large tumor size, microvascular invasion (MVI), multiple tumor nodules, and satellite lesions.^[4,14,16]^

Nomograms, as intuitive statistical models,^[17]^ can estimate the prognosis of individuals rather than groups and incorporate newly developed prognostic indicators.^[18]^ In various cancer types, nomograms predicted prognosis more accurate than conventional staging systems.^[19–22]^ Thus, it is considered as a more advanced tool for prognostic prediction.^[17]^

The aim of the present study was to develop a prognostic nomogram for individualized prognosis prediction in HCC patients underwent adjuvant TACE following curative resection. In addition, we compared the predictive accuracy of the proposed nomogram to conventional staging systems in terms of the concordance index (C-index) to ascertain whether they are accurate prognostic models.

## 2 Patients and methods

### 2.1 Patients

Data of 844 consecutive HCC patients who received curative resection from December 2010 to June 2012 in Zhongshan Hospital (Shanghai, People's Republic of China) were reviewed. The inclusion criteria were included as following: patients with any of the risk factors such as MVI, tumor size > 5 cm, multiple tumors and microsatellite lesions; no preoperative anticancer treatments; no history and concurrence of other malignant tumors; complete removal of macroscopic tumors; and received the first adjuvant TACE (1 to 3 courses; anthracycline, fluorouracil, platinum, and lipiodol) within 6 to 8 weeks after resection. The exclusion criteria were as follows: tumors with mixed types in histopathology or with uncertain origins; portal vein tumor thrombus; lymph node metastasis; distant metastasis before the operation; and incomplete follow-up data. Among the eligible patients, we randomly selected 144 patients as the training cohort and 86 patients as the validation cohort.

The clinical staging was based on the Barcelona Clinic Liver Cancer (BCLC) system,^[23]^ the American Joint Committee on Cancer (AJCC) 7th edition,^[24]^ and the Cancer of the Liver Italian Program (CLIP) score.^[25]^ Laboratory tests including liver function tests, blood routine, alpha-fetoprotein (AFP), and hyper-sensitive C-reactive protein (hs-CRP) were performed within 3 days before the surgery. Immunohistochemical staining of markers including cytokeratin 7 (CK7) and cytokeratin 19 (CK19) in the resected specimens was performed as a routine in the histopathologic analysis. CK7 and CK19 were considered positive if the staining tumor cells were more than 5%.^[26]^ The histologic grade of the tumor was defined by the Edmondson–Steiner classification.

This study was approved by research ethics committee of Zhongshan Hospital. The last follow-up was censored on November 2015. The informed consent was waived because of the retrospective nature of the study.

### 2.2 Follow-up

Postoperative follow-up was conducted every 1 to 3 months in the first year and every 3 to 6 months afterwards. The routine examinations according to the standard protocol included abdominal ultrasound, chest imaging examinations, and serum AFP. Suspected recurrences were further investigated by liver dynamic computed tomography (CT) or magnetic resonance imaging (MRI). The recurrence was diagnosed by typical imaging features in CT or MRI. The recurrence free survival (RFS) time was defined as time interval between the date of surgery and the date when recurrence was first identified. The overall survival (OS) was calculated from the date of surgery to death or the last follow-up. For patients without a documented OS/RFS event, the data were censored at the last follow-up. The median follow-up time was 38 months (range 2–54 months) in the training cohort and 30 months (range 3–60 months) in the validation cohort, respectively.

### 2.3 Statistical analysis

The identification of risk factors was performed by SPSS version 21.0 (SPSS, Chicago, IL). The distributions of both the OS and the RFS were depicted by the Kaplan–Meier method and analyzed by the log-rank test. Variables between 2 independent groups were compared using the Pearson Chi-squared test, Fisher's exact test, or the Mann–Whitney *U* test as appropriate. The cut-off value of a continuous variable was determined by the value with optimal Youden index after the receiver operating characteristic curve (ROC) was depicted. The Cox regression analysis was used for both univariate analyses and multivariate analyses. The multivariate model covariates were selected by a backward stepwise selection. The rms package in R project version 2.14.1 (http://www.r-project.org/) was used to establish the nomogram integrating variables that significantly related to OS in multivariate analyses. The discriminatory ability of the nomogram was quantified by the C-index. The calibration curve was used to identify the differences between the nomogram-predicted risks and the observed ones estimated by the Kaplan–Meier method. The decision curve analysis (DCA) was performed according to the online step-by-step tutorial provided by Vickers AJ et al.^[27,28]^

## 3 Results

### 3.1 Clinicopahtologic characteristics and prognosis of the patients

The clinicopathologic characteristics of the training cohort and the validation cohort are illustrated in Table 1.

**Table 1:**
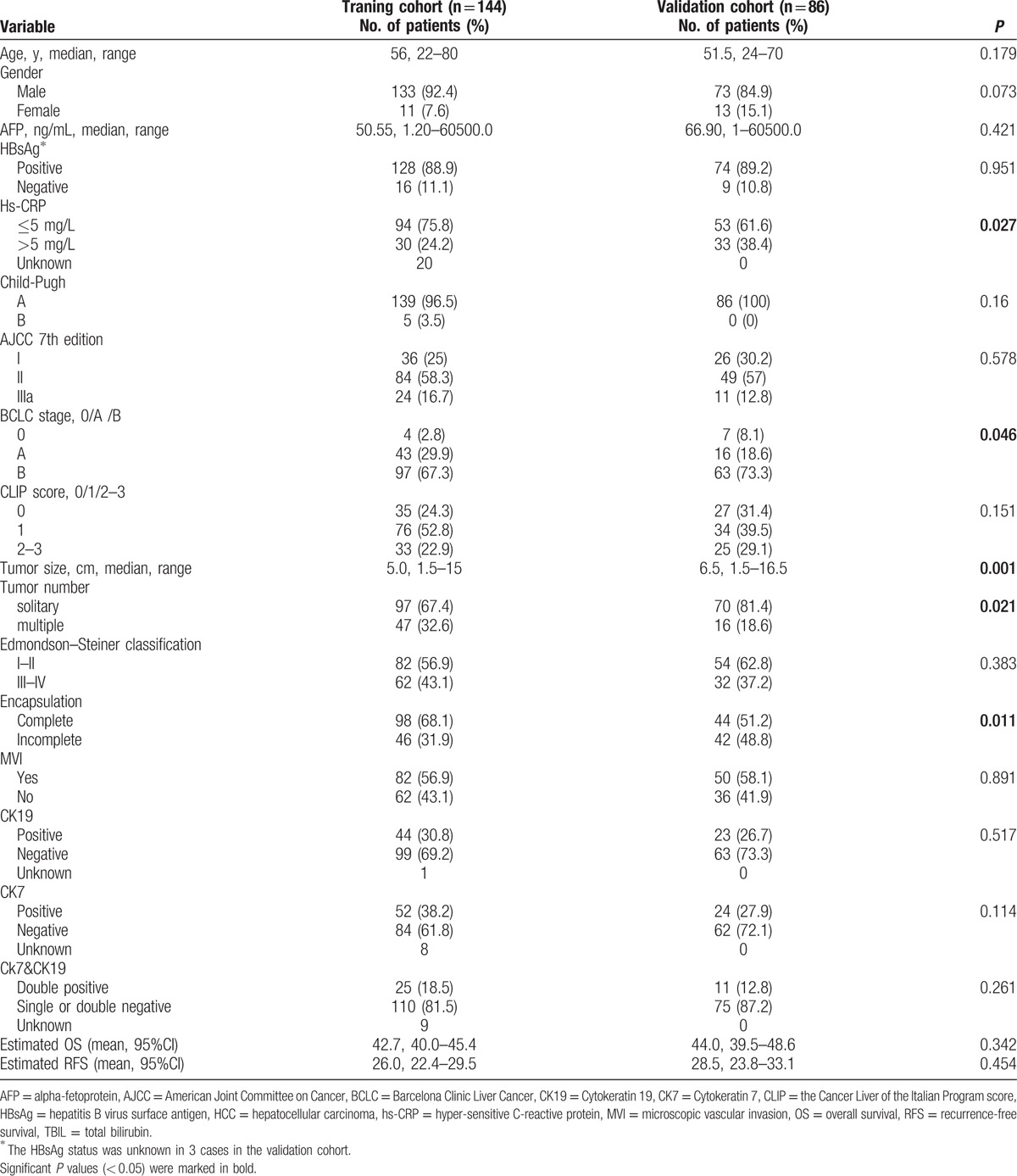
Clinicopathological characteristics of patients with HCC.

The 1-, 2-, 3-, and 4-year OS rates of the training cohort were 93.0%, 79.9%, 72.2%, and 64.8%, respectively. The 1-, 2-, 3-, and 4-year OS rates of the validation cohort were 87.2%, 74.3%, 68.2%, and 60.2%, respectively. The 1-, 2-, 3-, and 4-year RFS rates of the training cohort were 58.1%, 45.0%, 38.7 and 28.8%, respectively. The 1-, 2-, 3-, and 4-year RFS rates of the validation cohort were 66.0%, 43.4%, 37.0 and 34.4%, respectively.

The optimal cut-off value for hs-CRP was 4.4 and 5.6 mg/L for RFS and OS, respectively. Thus, a cut-off value of 5 mg/L was used in this study. Compared with the training cohort, the validation cohort included larger proportions of patients with hs-CRP > 5 mg/mL, with BCLC B stage, with incomplete tumor capsule, with larger tumor size, and with solitary tumor.

### 3.2 Independent prognostic factors for RFS and OS

In univariate analysis, the elevated serum AFP (*P*  < 0.001) and hs-CRP (*P* = 0.007**)** levels, AJCC 7th edition (*P* = 0.002), incomplete encapsulation of the tumor (*P* = 0.009), and MVI (*P* < 0.001) were identified as significant predictors for RFS. In multivariate analysis, the elevated AFP (*P* = 0.002, hazard ratio [HR] = 1.000, 95%CI, 1.000–1.000), hs-CRP levels (*P* = 0.029, HR = 1.756, 95%CI, 1.059–2.911), and MVI (*P* = 0.02, HR = 1.837, 95%CI, 1.102–3.061) remained independent risk factors for RFS (Table 2).

**Table 2:**
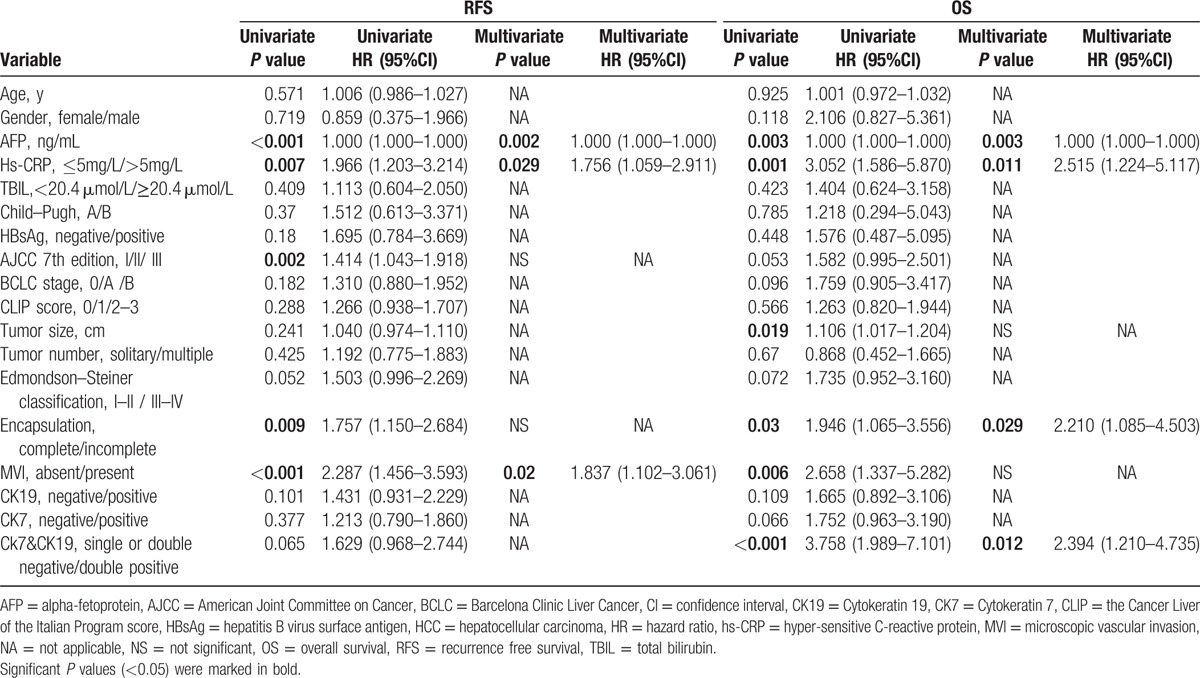
Clinicopathological characteristics of patients with HCC: univariate and multivariate analyses (training cohort).

The univariate analysis showed that the raised AFP (*P* = 0.003) and hs-CRP levels (*P* = 0.001), larger tumor size (*P* = 0.019), incomplete encapsulation of the tumor (*P* = 0.030), the presence of MVI (*P* = 0.006), and double positive staining for CK19 and CK7 (*P* < 0.001) to be significant predictors for OS. In multivariate analysis, raised AFP (*P* **=** 0.003, HR = 1.000, 95%CI, 1.000–1.000) and hs-CRP (*P* **=** 0.011, HR = 2.151, 95%CI, 1.224–5.117) levels, incomplete encapsulation of the tumor (*P* **=** 0.029, HR = 2.210, 95%CI, 1.085–4.503) positive staining for both CK19 and CK7 (*P* *<* 0.012, HR = 2.394, 95%CI, 1.210–4.735) were identified as independent risk factors for OS (Table 2).

### 3.3 Prognostic nomogram for OS

**Figure 1:**
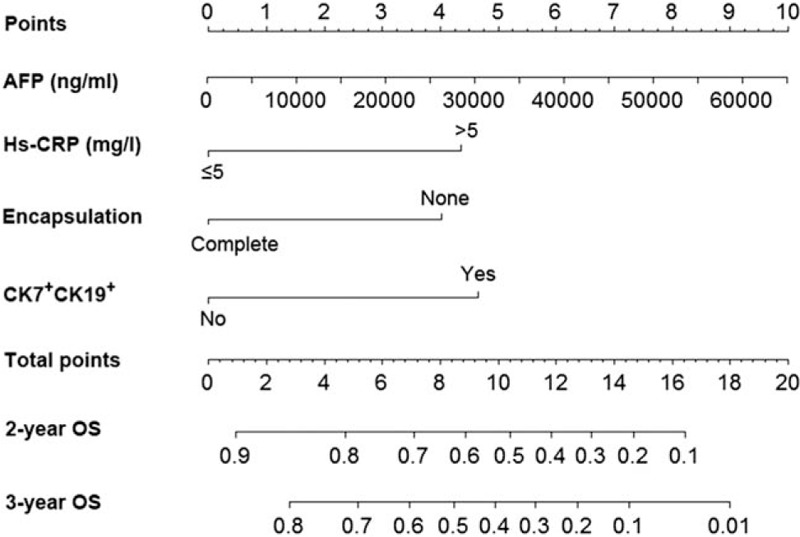
Prognostic nomogram for hepatocellular carcinoma patients underwent adjuvant transarterial chemoembolization after curative hepatectomy. To use the nomogram, first, a vertical line is drawn from the factor axis to the point scale to determine the number of points for each factor. Second, sum these numbers and locate it on the axis of the total points. Finally, draw a downward line from the axis of the total points to the survival axes to calculate the 2-year and 3-year survival probabilities. AFP = alpha-fetoprotein; CK7 = Cytokeratin 7; CK19 = Cytokeratin 19; hs-CRP = hyper-sensitive C-reactive protein.

The prognostic nomogram for OS prediction, integrating the significant independent risk factors in multivariate analyses for OS, is shown in Fig. 1.

**Figure 2:**
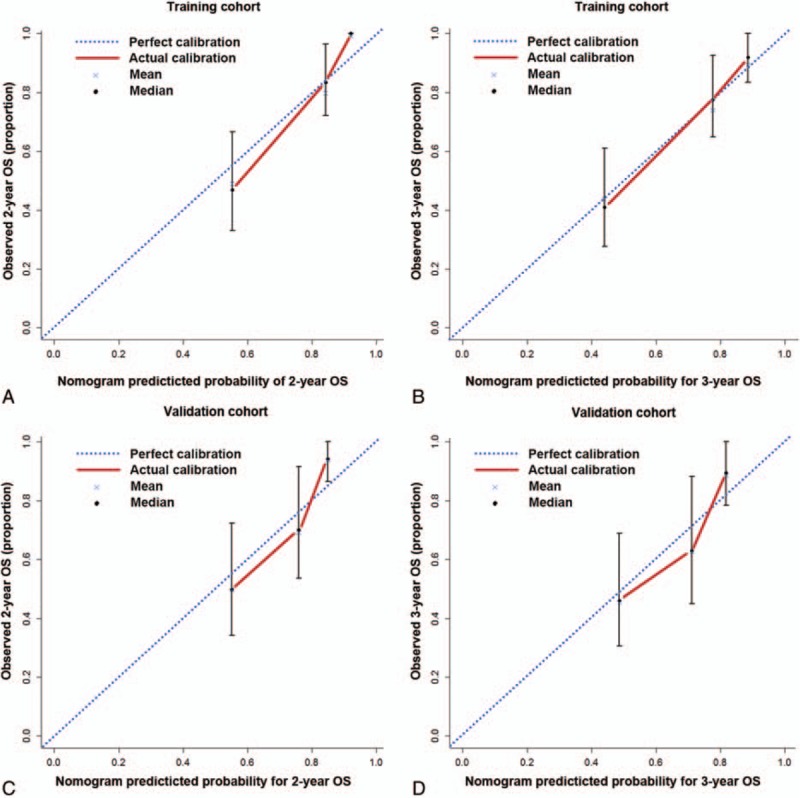
The calibration plots for predicting OS of patients at (A) 2 years and (B) 3 years in the training cohort; predicting OS of patients at (C) 2 years and (D) 3 years in the validation cohort. Nomogram-predicted probability of survival is plotted on the *x*-axis; observed survival is plotted on the *y*-axis. OS = overall survival.

The C-indices for the OS prediction were 0.787 (95% CI, 0.775–0.799). The calibration curves for the probability of OS at 2 and 3 years after surgery (Fig. 2A and B) showed optimal agreement between the nomogram-predicted probability and the actual observed probability.

### 3.4 Validation of the predictive performance of the nomogram for OS

In the validation cohort, the C-index of the constructed nomogram was 0.714 (0.695–0.733). The calibration curves showed good consistency between the observed and the nomogram predicted probability for 2-, 3-year OS (Fig. 2C and D) survival.

### 3.5 The comparison of nomogram with conventional staging systems

The comparative performance of the established nomogram and the conventional staging systems in terms of the C-index is listed in Table S1.

In the training set, the C-indices of the BCLC staging, the AJCC 7th edition and the CLIP score for OS prediction were 0.553 (95% CI, 0.542–0.564), 0.593 (95% CI, 0.581–0.604), and 0.556 (95% CI, 0.542–0.569), respectively. In the validation set, the C-indices of the BCLC staging, the AJCC 7th edition and the CLIP score for OS prediction were 0.638 (95% CI, 0.626–0.650), 0.558 (95% CI, 0.539–0.577), and 0.571 (95% CI, 0.552–0.590), respectively.

Taken together, the constructed nomogram displayed superior predictive accuracy compared with the conventional staging systems in both the training cohort and the validation cohort.

**Figure 3:**
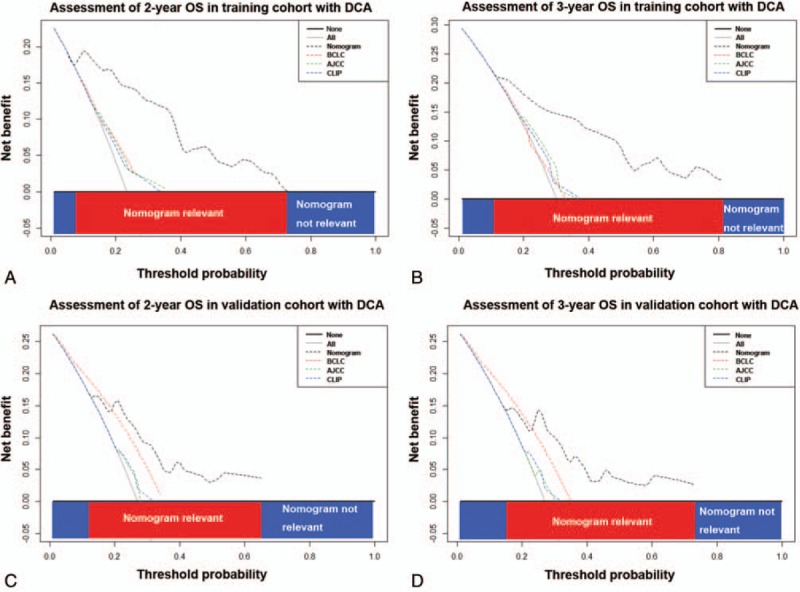
Decision curve analysis for the nomogram and the conventional staging systems, including the BCLC staging system, the AJCC 7th edition and the CLIP score. The nomogram is compared with the conventional staging systems in terms of 2-year (A for training cohort; C for validation cohort) and 3-year OS (B for training cohort; D for validation cohort), respectively. Dashed lines: clinical net benefits across a range of threshold probabilities; the horizontal solid black line: to assume no patients will experience the event; the solid gray line: to assume all patients will experience the event. On decision curve analysis, the nomogram yielded superior clinical net benefit compared with the conventional staging systems across a range of threshold probabilities. AJCC = American Joint Committee on Cancer, BCLC = Barcelona Clinic Liver Cancer, CLIP = the Cancer Liver of the Italian Program, OS = overall survival.

The DCA, a novel method to evaluate prediction models from the perspective of clinical consequences, revealed that, compared with the conventional staging systems, the nomogram yielded superior net clinical benefit across a wider range of threshold probabilities for predicting 2- and 3-year OS in the training cohort (Fig. 3A and B) and 2- and 3-year OS in the validation cohort (Fig. 3C and D), respectively.

### 3.6 Predictive performance of the nomogram in HCC patients with BCLC B stage

The discriminatory ability of the nomogram in HCC patients with BCLC B stage in the training cohort and validation cohort evaluated by C-indices were 0.782 (95% CI, 0.763–0.801) and 0.678 (95% CI, 0.653–0.702), respectively.

**Figure 4:**
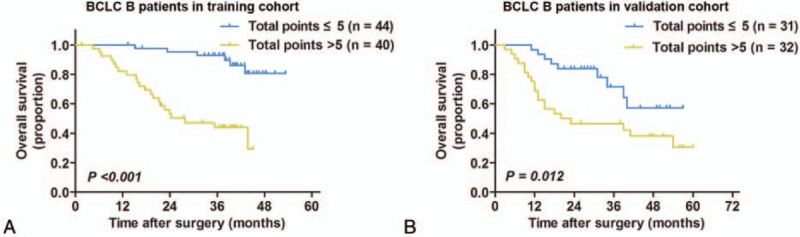
Kaplan–Meier survival curves for HCC patients with BCLC B stage who received adjuvant transarterial chemoembolization following curative resection stratified by total points calculated by the nomogram in the training cohort (A) and validation cohort (B). BCLC B patients with nomogram-calculated total points > 5 were associated with significant unfavorable OS compared with patients whose total points  ≤ 5. BCLC = Barcelona Clinic Liver Cancer, HCC = hepatocellular carcinoma.

The optimal cut-off values of the total points calculated by the nomogram in the BCLC B stage patients in the training set and the validation set were 4.7 and 5.2, respectively. Herein, the patients with BCLC B stage HCC were dichotomized into 2 subgroups with total points > 5 and ≤ 5, respectively. As shown in Fig. 4, in HCC patients with BCLC B stage, nomogram calculated total points > 5 were associated with poorer survival (*P* < 0.001 in the training set; *P* = 0.012 in the validation set).

## 4 Discussion

In this study, we established a prognostic nomogram integrating independent prognostic predictors, ranging from tumor biological markers to the clinicopathological features, for HCC patients underwent adjuvant TACE following curative resection. In terms of discriminatory ability, the C-index of the nomogram was significantly higher than that of BCLC staging system, the AJCC 7th edition and the CLIP score. Furthermore, the DCA showed that the nomogram yielded superior net benefit in clinical use compared with the conventional staging systems in this subgroup of HCC patients.

It is noteworthy that a large proportion of patients in this study were within BCLC B stage which may hamper the predictive performance of the BCLC staging system. Although the BCLC therapeutic flowchart recommended TACE rather than resection as initial treatment for patients with BCLC B stage, several studies suggested expanding the indication of surgical resection into BCLC B stage patients with well-preserved liver function.^[29–31]^ Recently, a survey conducted by the Italian Liver Cancer group suggested that TACE was not the optimal treatment for BCLC B stage patients and some patients within this stage were suitable for more aggressive treatments such as resection.^[31]^ Patients with BCLC B stage who underwent resection as initial treatment were more likely to meet the indications of adjuvant TACE for its overlapping elements with the BCLC B stage, such as larger tumor size and multiple tumors. Therefore, adjuvant TACE was more frequently performed in these patients. Taken together, there was a paucity of information on survival prediction for the subgroup of HCC patients underwent adjuvant TACE following curative resection, among which a large portion of patients were within BCLC B stage. To address this concern, we established a prognostic nomogram to offer additional information in stratifying the prognosis for HCC patients. Moreover, the constructed nomogram performed well within patients with BCLC B stage patients and BCLC B patients with the nomogram calculated total points > 5 showed significant poorer survival.

Recently, an increasing number of novel prognostic predictors, such as elements of tumor biology^[32]^ and indicators of systemic inflammation,^[18,33]^ were proposed for a better survival prediction in HCC. AFP was a routinely monitored tumor marker in HCC patients; higher AFP may suggest more advanced tumor burden, more aggressive phenotype, and higher risk of residual tumors after surgery.^[14,25]^ Preoperative AFP has been incorporated in several prognostic nomograms for HCC as either a categorical variable or after a logarithmic transformation.^[19,34,35]^ In this study, the AFP level was incorporated as a continuous variable because of its wide range in this cohort and its positive correlation with poor survival.

Inflammation is an emerging hallmark of cancer.^[36]^ CRP, an acute-phase inflammatory reactant synthesized in hepatocytes, is a key component in inflammation based prognostic score systems for HCC.^[37–39]^ The cut-off value of 10 mg/L for CRP was set empirically in most studies.^[37,38,40]^ Of note, hs-CRP, more sensitive and accurate than CRP, has different limits of quantification.^[41]^ Liu et al^[42]^ proposed a cut-off value of 4 mg/L for hs-CRP in the risk stratification of postoperative HCC patients, which is close to our cut-off value. Since routine CRP quantification methods are gradually replaced by automated hs-CRP methods,^[41]^ further studies to determine an optimal hs-CRP cut-off value for outcome prediction in HCC are warranted.

MVI was a proposed indication for postoperative adjuvant TACE in HCC patients.^[14,16]^ Consistent with the present study, several studies reported MVI as an independent risk factor for prognosis and incorporated it into prognostic nomograms.^[19,34,35,43,44]^ Nonencapsulated HCC was prone to direct liver invasion, tumor microsatellite formation, and vascular invasion.^[45]^ Unfavorable outcomes were observed among patients with nonencapsulated HCC in most studies, which were in accordance with our results.^[45–48]^

CK7 and CK19 were biomarkers for hepatic progenitor cells and cholangiocytes.^[26]^ Several studies have shown that positive CK19 to be a predictor for early recurrence and poor survival in HCC.^[26,32,49,50]^ In this study, CK19 was not identified as a significant predictor for both RFS and OS in univariate analysis. However, when the RFS and OS were censored at 2 years, CK19 was a significant risk factor for recurrence and poor survival (*P* = 0.024 and *P* = 0.028, for RFS and OS, respectively; Table S2). Moreover, our previous study identified overexpression of CK7 and CK19 in HCC patients with recurrence and developed a predictive model with both variables included.^[49]^ Thus, it is logic to combine CK19 with CK7 for a more accurate prediction. In addition, considering the chemo-resistant nature of progenitor cells^[51]^ and unfavorable improvement of adjuvant TACE in HCC with a high progenitor cell profile,^[49]^ other adjuvant treatment strategies that outperformed adjuvant TACE are eagerly participated for CK7/CK19 double positive HCC.

The limitations of this study are listed as following: first, the nomogram was established based on a retrospective, single-institutional cohort. Thus, the performance of this nomogram in patients from other institution, with different geographic and disease background, or in a prospective setting remains to be validated. In addition, a cut-off point for treatment decision making should be explored in future studies. In spite the shortcomings mentioned above, the established nomogram may offer input to optimize monitoring strategies for postoperative HCC with recurrence risk factors underwent adjuvant TACE.

In conclusion, we developed easy-to-use prognostic nomogram for patients underwent adjuvant TACE following curative resection, a special HCC subgroup in which, according to the proposed indications for adjuvant TACE, the majority of the participants were postoperative BCLC B stage patients. Compared to traditional staging systems, this nomogram incorporated indicators of systemic inflammation and tumor biology and performed better in prognostic prediction.

## Supplementary Material

Supplemental Digital Content
