# Supplementary material for: Prognostic nomogram for patients with hepatocellular carcinoma underwent adjuvant transarterial chemoembolization following curative resection
Source: Medicine (Baltimore). 2017 Mar 24;96(11):e6140. doi: 10.1097/MD.0000000000006140 (PMC5369882; doi:10.1097/MD.0000000000006140)
Supplement: Supplemental Digital Content [file medi-96-e6140-s001.doc]

| **Table S1. C-indices of the nomogram and conventional staging systems for prediction of OS.** | | | | | |
| --- | --- | --- | --- | --- | --- |
| **Predictive models** | **Training cohort** | |  | **Validation cohort** | |
|  | **C-index** | **95%CI** |  | **C-index** | **95%CI** |
| **Nomogram** | 0.787 | 0.775 - 0.799 |  | 0.714 | 0.695 - 0.733 |
| **BCLC stage** | 0.553 | 0.542 - 0.564 |  | 0.638 | 0.626 - 0.650 |
| **AJCC 7th edition** | 0.593 | 0.581 - 0.604 |  | 0.558 | 0.539 - 0.577 |
| **CLIP** | 0.556 | 0.542 - 0.569 |  | 0.571 | 0.552 - 0.590 |
| Abbreviations: BCLC = Barcelona Clinic Liver Cancer; AJCC = American Joint Committee on Cancer; CLIP = the Cancer Liver of the Italian Program score; OS, overall survival; CI, confidence interval. | | | | | |

| **Table S2. Log-rank tests for positive staining of CK19 censored at different time after surgery for RFS/OS.** | |
| --- | --- |
| **Censored time** | ***P*-value** |
| **RFS, year** |  |
| **2** | **0.024** |
| **3** | 0.076 |
| **4** | 0.098 |
| **OS, year** |  |
| **2** | **0.028** |
| **3** | **0.036** |
| **4** | 0.105 |
| Abbreviations: OS = overall survival; RFS = recurrence free survival; CK19 = Cytokeratin 19; Significant P values (<0.05) were marked in bold. | |
